# Persistent Spatial Clusters of Prescribed Antimicrobials among Danish Pig Farms – A Register-Based Study

**DOI:** 10.1371/journal.pone.0136834

**Published:** 2015-08-28

**Authors:** Mette Fertner, Javier Sanchez, Anette Boklund, Henrik Stryhn, Nana Dupont, Nils Toft

**Affiliations:** 1 Section for Epidemiology, National Veterinary Institute, Technical University of Denmark, Frederiksberg, Denmark; 2 Centre of Veterinary Epidemiological Research, Department of Health Management, Atlantic Veterinary College, University of Prince Edward Island, Charlottetown, Canada; 3 Department of Large Animal Sciences, University of Copenhagen, Frederiksberg, Denmark; Wageningen University and Research Centre, NETHERLANDS

## Abstract

The emergence of pathogens resistant to antimicrobials has prompted political initiatives targeting a reduction in the use of veterinary antimicrobials in Denmark, especially for pigs. This study elucidates the tendency of pig farms with a significantly higher antimicrobial use to remain in clusters in certain geographical regions of Denmark. Animal Daily Doses/100 pigs/day were calculated for all three age groups of pigs (weaners, finishers and sows) for each quarter during 2012–13 in 6,143 commercial indoor pig producing farms. The data were split into four time periods of six months. Repeated spatial cluster analyses were performed to identify persistent clusters, i.e. areas included in a significant cluster throughout all four time periods. Antimicrobials prescribed for weaners did not result in any persistent clusters. In contrast, antimicrobial use in finishers clustered persistently in two areas (157 farms), while those issued for sows clustered in one area (51 farms). A multivariate analysis including data on antimicrobial use for weaners, finishers and sows as three separate outcomes resulted in three persistent clusters (551 farms). Compared to farms outside the clusters during this period, weaners, finishers and sows on farms within these clusters had 19%, 104% and 4% higher use of antimicrobials, respectively. Production type, farm type and farm size seemed to have some bearing on the clustering effect. Adding these factors as categorical covariates one at a time in the multivariate analysis reduced the persistent clusters by 24.3%, 30.5% and 34.1%, respectively.

## Introduction

In Denmark, 29 million pigs are produced annually accounting for 76% of prescribed veterinary antimicrobials [1]. There has been an increase in public awareness surrounding the prudent use of veterinary antimicrobials due to the emergence of antimicrobial resistance [2,3,4]. Subsequently, a number of legislative actions targeting a reduction in the use of antimicrobials for pigs have been launched in Denmark [5,6,7,8].

Antimicrobial treatment of production animals is, according to Danish legislation, restricted to clinical disease, thus excluding use for prophylaxis and growth promotion [9]. In Denmark, the three age groups of pigs, for which antimicrobials are prescribed are: weaners, finishers and sows (including boars and piglets). The consistency in the overall antimicrobial consumption at a farm is therefore ideally assessed using a multivariate analysis combining the use in all three groups simultaneously. The primary clinical reasons for prescribing antimicrobials are gastrointestinal and respiratory disorders for weaners and finishers, and limbs/joints/CNS/skin and urogenital disorders for sows [10]. Management and medication practices vary substantially among Danish pig farmers. The choice of drug, dose and treatment time as well as the perception of metaphylaxis all influences the administration of antimicrobials at the farm.

For sow farms, densely populated areas have been found to have a higher use of antimicrobials than sparsely populated areas [11]. Furthermore, the amount of antimicrobials prescribed for gastrointestinal disorders in finishers has been found to be highly affected by geographical region [12]. Additionally, treatment practices on farm has been shown to remain stable over time [13]. Due to variation in farm density, veterinary affiliation and a presumed stability in treatment practices on farm, our hypothesis was that a number of persistent spatial clusters exist in the amount of antimicrobials prescribed for pigs. Thus, the objective of this study was to identify and characterize the spatial clusters of Danish indoor commercial pig producing farms that persistently prescribed significantly more antimicrobials during 2012–13.

## Materials and Methods

### Study design

**Fig 1:**
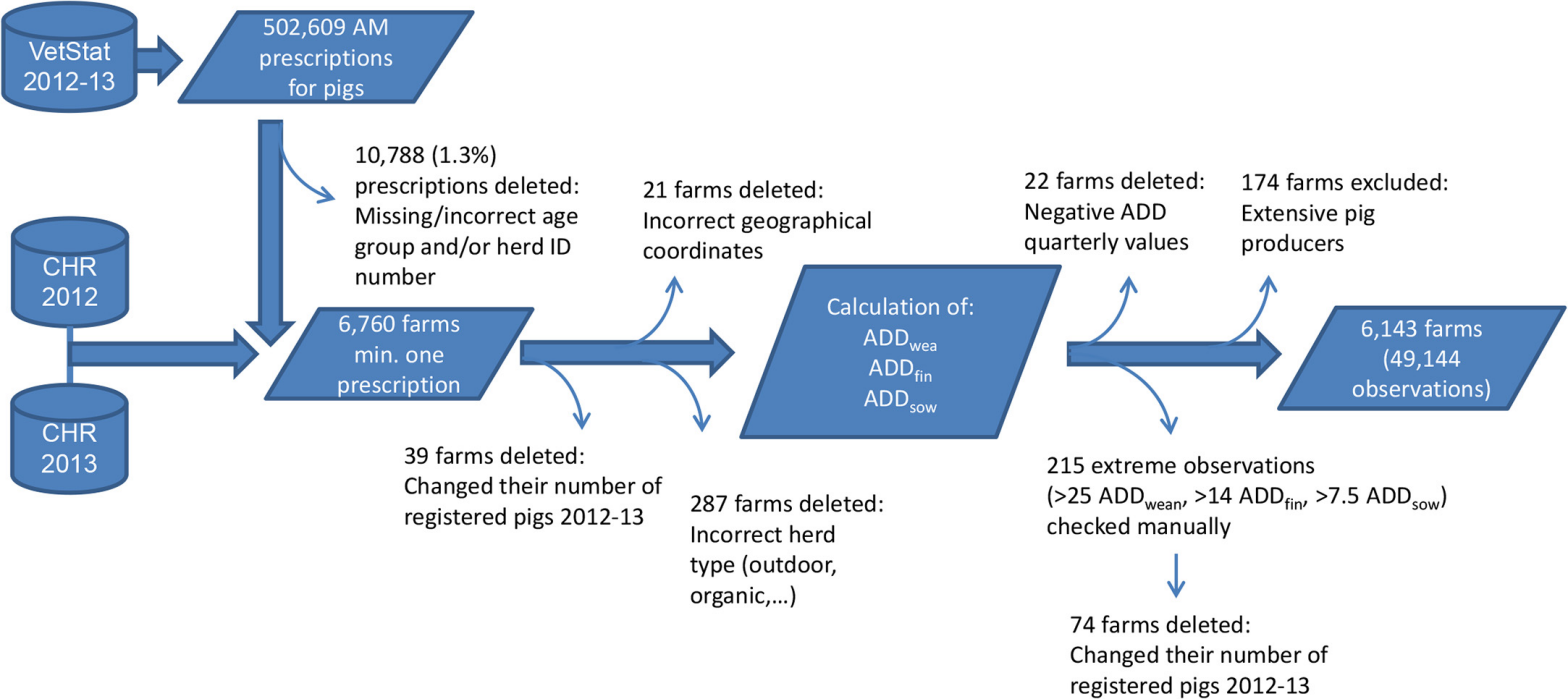
Flow diagram of data management. Data extracted from the Danish national databases VetStat and CHR were used to calculate up to three standardized measures of antimicrobials for each pig farm (ADD_wea_, ADD_fin_ and ADD_sow_). AM = antimicrobials.

The study was designed as a register-based study on antimicrobial use during the years 2012 and 2013. Data from all indoor commercial pig farms were included in the study, with the exception of those excluded due to recording mistakes (Fig 1).

### Study population

A total of 6,143 farms were included in the study population and were characterized in terms of their Cartesian coordinates for geographic location, farm type (production/nucleus), type of production (presence of one or more age groups), farm size (number of pigs, separately evaluated for each of the three age groups) and Specific Pathogen Free (SPF) status. To insure that only active farms were included in the study, a minimum of one prescription during the two-year study period was required (6,760 farms). Reasons for exclusion were missing (19 farms) or identical Cartesian coordinates (2 farms), changes in farm size (39 farms), farm type (e.g. outdoor, organic, boar stations) (287 farms), negative antimicrobial values in one of the quarters (22 farms) and extensive pig producers (174 farms) with fewer than 50 sows, 200 finishers and 200 weaners (Fig 1).

Production farms (5,915 participating) are defined as farms producing weaners and/or finishers, while nucleus farms (228 participating) are defined as farms only producing breeding stock [14]. Farms participating in the voluntary SPF system have a certain level of biosecurity and are aware of the on-farm infection status of two specified ectoparasites and five pathogens [15]. Data from the SPF system were extracted in March 2013, while data on farm demographics were retrieved from the Central Husbandry Register (CHR) in January 2012 and October 2013.

### Antimicrobial prescription

The quantity of antimicrobials prescribed at a given farm was assumed to be a consistent proxy for the level of consumed antimicrobials during the given time period. Since 2000, all veterinary antimicrobial prescriptions for production animals have been recorded in the national Danish database, VetStat [16]. VetStat receives information from three sources: Feed mills, veterinarians and pharmacies. For pigs, more than 98% of the total number of prescriptions for pigs is recorded by pharmacies. To avoid the influence of legislative initiatives [17,18], we did not include data prior to January 2012. This study used prescriptions issued by pharmacies from the period of 1^st^ January 2012 to 31^st^ December 2013; in total 844,704 prescriptions of where 502,609 were antimicrobial prescriptions (Fig 1). Registrations were retrieved from VetStat on 31^st^ March 2014. Each prescription contained detailed information on the prescription date, prescribing veterinarian, recipient (farm number), animal species, age group, clinical indication, antimicrobial product and amount of antimicrobial [16,10]. However, 10,788 (1.3%) prescriptions were deleted due to a missing (or incorrect) age group (10,674) and/or farm identification number (132).

Antimicrobials were assessed as Animal Daily Doses (ADD). One ADD is defined as the dose needed to treat one pig of a given size for one day for the main indication. VetStat uses standard weights for treatment in each of the three age categories: 15 kg (weaners), 50 kg (finishers) and 200 kg (sows, boars and piglets). One ADD_15_ equals one standard dose needed to treat one standard weaner (15 kg pig) for one day. Likewise ADD_50_ and ADD_200_ are calculated for finishers and sows. The number of ADDs aggregated on the farm level for each of the three age groups was divided by the number of pig days at the farm. The number of registered pigs in each of the three age groups was extracted from the CHR register and multiplied by the number of days in the given time period, to calculate the total number of pig days at risk. This standardized unit is consistent with the official unit: Prescribed number of ADD per 100 pigs per day (ADD/100 pigs/day), which approximates the percentage of pigs treated at the farm daily [7,19]. Therefore, up to three estimates were calculated per farm: ADD_15_/100 weaners/day, ADD_50_/100 finishers/day and ADD_200_/100 sows/day, denoted here as ADD_wea_, ADD_fin_ and ADD_sow_, respectively. These standardized measures enable comparison across farms, despite variations in farm size and choice of drug [19]. In VetStat, each antimicrobial product was initially assigned an appropriate dose based on pharmaceutical approval. In 2014, the doses were revised [7]. For this project, results are presented using the new doses.

Most pig farms have a health counseling contract, which includes visits by a veterinarian 9–12 times a year (4–6 times a year for finisher-only farms) [9]. Drugs are typically prescribed in connection with such visits. In support of this, Vigre et al [20] identified the median duration of prescription period to be 36 days for weaners and 39 days for finishers. Therefore, prescribed antimicrobials were aggregated quarterly for each of the three age groups to reflect the actual use of antimicrobials within a given time period.

The three right-skewed continuous distributions, ADD_wea_, ADD_fin_ and ADD_sow_, were log-transformed to reduce the influence of extreme values. A relatively large number of the observations were zero (13% sows, 19% weaners and 28% finishers). To allow transformation despite the observations of zero, a small constant was added to the total amount of prescribed antimicrobial at each farm. This constant was added prior standardization and transformation, so that a zero observation in a large farm was assigned a smaller value than a zero observation in a small farm. The added value equaled half the smallest amount of prescribed antimicrobials during the first quarter corresponding to 29 ADD_15_ for weaners, 6.5 ADD_50_ for finishers and 1 ADD_200_ for sows.

### Spatio-temporal analyses

The scan statistic can be used in the identification of local clustering of an event in space and time. Traditionally, the procedure has been used to investigate clustering of disease in human as well as veterinary epidemiological studies. Scan statistic is based on a circular scanning technique using the log likelihood ratio test [21]. Recently, the univariate scan statistic has been extended to include continuous outcomes [22] and may incorporate multiple datasets (e.g. different diseases or different population characteristics) [23]. This multivariate scan statistic method has so far only been sparsely applied in veterinary epidemiology [24]. To our best knowledge, this is the first study in veterinary epidemiology to make use of a multivariate scanning technique with a continuous outcome.

Here, we made use of both the scan statistic methods (univariate and multivariate) to test whether the mean of ADD_wea_, ADD_fin_ and/or ADD_sow_ in Danish commercial indoor pig farms was higher in certain geographical areas throughout time than would be expected due to chance. To allow for unrestricted geographical overlap of clusters in different time periods, repeated spatial analyses were performed, rather than a single spatio-temporal analysis. To increase the study power the scan statistic was ran on a six-month scale; hence, each analysis included two observations per age group at a farm, one for each quarter. The geographical areas included in a significant cluster in all four consecutive time periods were defined as a persistent cluster. Following this, the total number of farms within the intersection of the four significant clusters was identified.

Purely spatial retrospective analyses were executed using a normal probability model [22]. Initially, univariate models were run for each of the three outcomes separately: Ln(ADD_wea_), ln(ADD_fin_) and ln(ADD_sow_). Subsequently, by including all three datasets (ln(ADD_wea_), ln(ADD_fin_) and ln(ADD_sow_)), a multivariate version of the model [23] was used. Additionally, three categorical covariates (production type (7 levels), farm type (2 levels) and farm size (3 levels)) were added one at a time to the multivariate model, in order to investigate the effect on clustering.

The maximum spatial cluster size was set to 20% (1,229 farms) of the population at risk. No geographical overlap was allowed in the individual analyses. An elliptic spatial shape was selected to account for edge effects of the estimated cluster areas. However, the exact borders of the underlying true clusters remain uncertain regardless of the shape used [25].

For each of the generated elliptic windows (e.g. scanning windows) around each location, the log-likelihood for observing a higher mean ADD value within the window was calculated. The window with the maximum log-likelihood was identified as the most likely (or primary) cluster. The distribution of log-likelihood ratio statistic under the null hypothesis was evaluated using Monte Carlo hypothesis testing. In the multivariate analysis, clustering which occurred in a single dataset or in more datasets simultaneously was evaluated. This was achieved by establishing a combined log likelihood defined as the sum of log likelihoods from each of the individual datasets where the observed antimicrobial use exceeded the expected use [23]. Significant ellipses (p<0.05) contributing to a persistent cluster were plotted on a map. A chi-square test was applied to test whether farm characteristics were significantly different inside compared to outside the clusters.

Data management was carried out using the software SAS [26]. Subsequently, data were exported to R [27], for statistical analysis. Spatial analysis was carried out in SaTScan [28].

## Results

**Fig 2:**
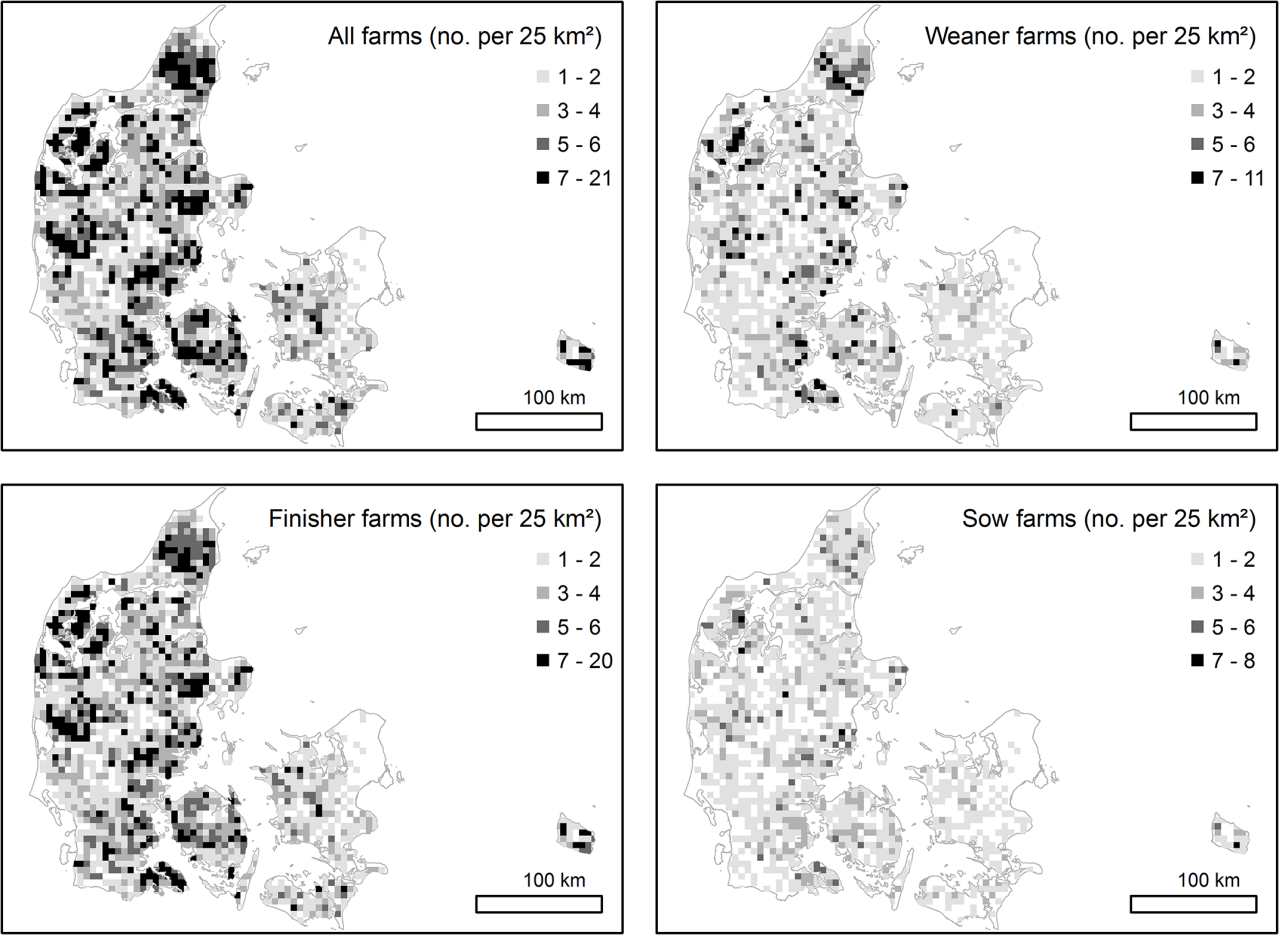
Map of the Danish indoor commercial pig farm density. “All farms” illustrate the geographical distribution of all 6,143 indoor commercial pig farms in Denmark. Maps denoted “weaner farms” (2,886), “finisher farms” (5,417) and “sow farms” (2,062) illustrate farms holding the respective age groups. Colors indicate number of farms present in each square (5*5 kilometer).

As illustrated in Fig 2, the farm density of participating farms is generally higher in the western part of Denmark than the eastern part.

Based on data from the CHR and from the SPF register, farms included in the analyses are described by their SPF status, production type, and farm size (Table 1).

**Table 1:** Characteristics of 6,143 Danish indoor commercial pig producing farms, based on data from 2012–13.

Farm characteristics		Numbers of farms (%)
**Conventional vs SPF status**	Conventional farms (non-SPF)	3,419
	Farms in SPF register	2,724
**Production type(age groups present at the farm)**	Farrow-to-finisher	1,430 (23.3)
	Sows and finishers	159 (2.6)
	Sows and weaners	288 (4.7)
	Sows	185 (3.0)
	Weaner and finishers	915 (14.9)
	Weaners	253 (4.1)
	Finishers	2,913 (47.4)
**Farm size**		
Presence of sows*	Small (1–254)	513 (24.9)
	Medium (255–670)	1,043 (50.6)
	Large (> 670)	506 (24.5)
Presence of weaners*	Small (1–783)	722 (25.0)
	Medium (784–2300)	1,458 (50.5
	Large (> 2300)	706 (24.5)
Presence of finishers*	Small (1–499)	1,342 (24.8)
	Medium (500–1600)	2,769 (51.1)
	Large (> 1600)	1,306 (24.1)

*One farm may appear in multiple categories of farm size if more age groups are present at the farm.

Prescribed antimicrobials standardized as ADD_wea_, ADD_fin_ and ADD_sow_, for each quarter of 2012 and 2013 in the 6,143 study farms are presented in (Table 2). Depending on the production type, each farm held information on up to three measurements of antimicrobial consumption (one for each age group of pigs). The variable ‘production type’ had seven levels, defining the presence or absence of the three age groups of pigs. Farm size was categorized according to the quartiles for each of the three age groups: Small (<Q1), Medium (Q1 –Q3) and Large (>Q3). Information on farm type, production type and farm size were complete for all 6,143 farms and were used as covariates in the multivariate model.

**Table 2:** Median and interquartile ranges of the amount of prescribed antimicrobials for three ages groups for each quarter in 2012–13 in 6143 Danish indoor commercial pig producing farms.

		Weaners	Finishers	Sows
		ADD_wea_ [IQR]	ADD_fin_ [IQR]	ADD_sow_ [IQR]
2012	Jan–Mar	8.47 [2.72;14.64]	0.97 [0.04;2.88]	1.92 [1.07;2.98]
	April–Jun	8.05 [2.06;14.63]	0.76 [0.00;2.66]	1.77 [0.96;2.78]
	Jul–Sep	7.44 [1.70;13.61]	0.76 [0.00;2.65]	1.69 [0.88;2.68]
	Oct–Dec	8.17 [1.76;14.40]	0.87 [0.00;2.79]	1.75 [0.88;2.75]
2013	Jan–Mar	8.10 [1.25;15.43]	0.82 [0.00;2.97]	1.92 [0.86;3.03]
	April–June	7.93 [0.99;14.92]	0.70 [0.00;2.75]	1.87 [0.81;2.99]
	Jul–Sep	7.53 [0.69;14.37]	0.69 [0.00;2.77]	1.79 [0.76;2.90]
	Oct–Dec	8.18 [1.12;14.83]	0.98 [0.00;3.12]	1.84 [0.75;2.91]

ADD = Animal Daily Doses per 100 animals per day.

**Fig 3:**
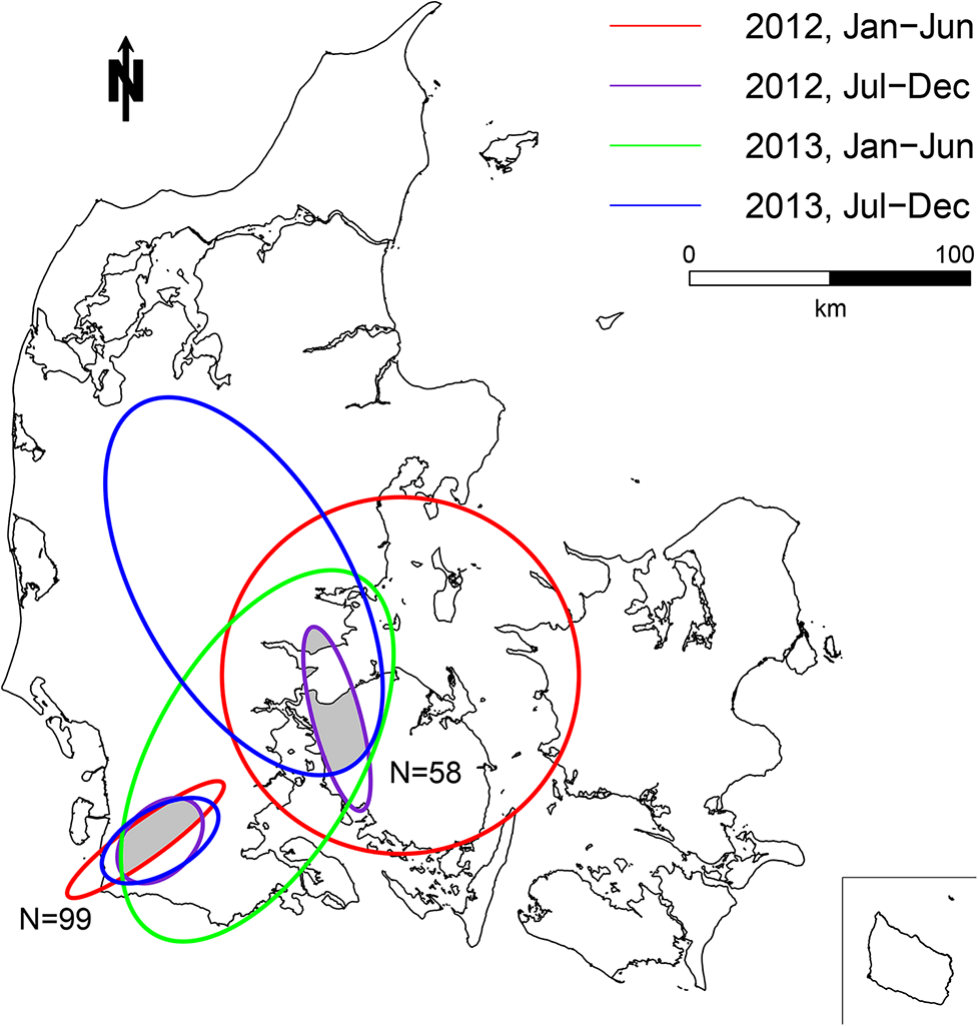
Map of the univariate persistent clusters of antimicrobials prescribed for finishers. Each ellipse illustrates a significant cluster (p<0.05) in one of the four time periods. Two persistent clusters were identified, including a total of 157 farms. N indicates the number of farms inside each of the persistent clusters.

**Fig 4:**
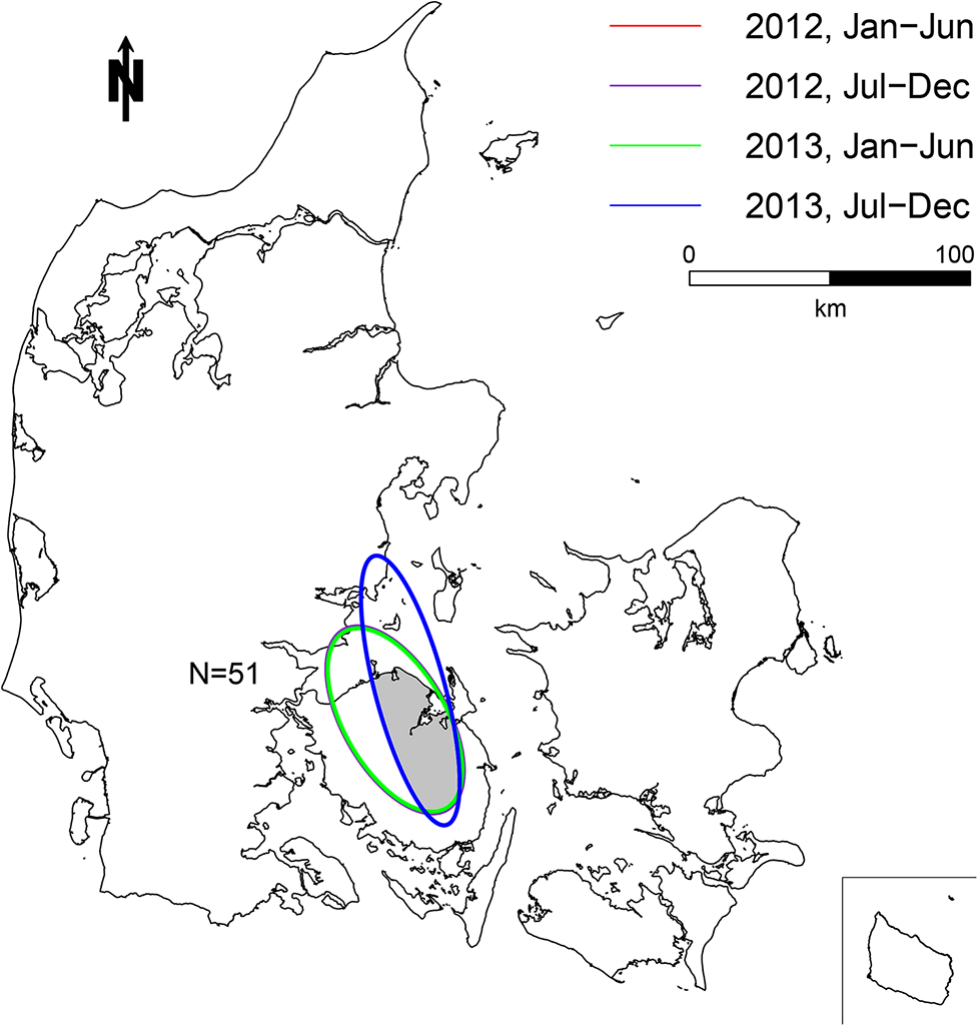
Map of the univariate persistent cluster of antimicrobials prescribed for sows. 51 farms located in one persistent cluster area. Each ellipse illustrates a significant cluster (p<0.05) in one of the four time periods. The three significant clusters from January 2012 to June 2013 lie on top of each other, which is why only two ellipses are visible. N indicates the number of farms inside the persistent cluster area.

Two persistent clusters were identified by the univariate cluster analysis on antimicrobials prescribed for finishers. These clusters included 99 and 58 farms, respectively (Fig 3). On average, finishers inside these persistent clusters consumed 158% more antimicrobials (2.01 ADD_fin_) than finishers outside the clusters (0.78 ADD_fin_) (Table 3). Likewise, a univariate model on antimicrobials prescribed for sows resulted in one persistent cluster of 51 sow farms (Fig 4) consuming 38% more antimicrobials (2.49 ADD_sow_) compared to sow farms outside the clusters (1.80 ADD_sow_) (Table 3). The univariate analysis on antimicrobials prescribed for weaners did not result in any persistent clustering.

**Table 3:** Median and interquartile ranges for antimicrobial use in 6,143 Danish pig farms inside and outside identified persistent clusters from 2012–13.

		No. of farms	ADD_wea_[IQR]	ADD_fin_ [IQR]	ADD_sow_[IQR]
All farms		6,143	7.99 [1.51;14.59]	0.82 [0.00;2.82]	1.82 [0.88;2.87]
**Univariate models**					
Antimicrobial for sows	Inside	51			2.49 [1.54;3.64]
	Outside	2,011			1.80 [0.87;2.86]
Antimicrobial for finisher	Inside	157		2.01 [0.52;4.21]	
	Outside	5,260		0.78 [0.00;2.78]	
**Multivariate model**	Inside	551	9.40 [3.97;15.93]	1.55 [0.13;3.57]	1.89 [1.06;2.94]
	Outside	5,592	7.88 [1.30;14.48]	0.76 [0.00;2.73]	1.81 [0.86;2.87]
+ Farm type*	Inside	383	10.47 [4.52;16.76]	1.74 [0.14;3.86]	1.97 [0.96;3.04]
	Outside	5,760	7.87 [1.38;14.47]	0.76 [0.00;2.74]	1.81 [0.88;2.87]
+ Production type*	Inside	417	9.97 [4.62;16.46]	1.55 [0.14;3.56]	1.87 [1.08;2.84]
	Outside	5,726	7.88 [1.30;14.48]	0.77 [0.00;2.75]	1.81 [0.87;2.88]
+ Farm size*	Inside	363	10.94 [5.09;17.16]	1.81 [0.18;3.94]	1.89 [0.99;2.94]
	Outside	5,780	7.87 [1.35;14.45]	0.77 [0.00;2.74]	1.81 [0.88;2.87]

*The regular multivariate model added one of the three covariates separately (farm type, production type or farm size).

**Fig 5:**
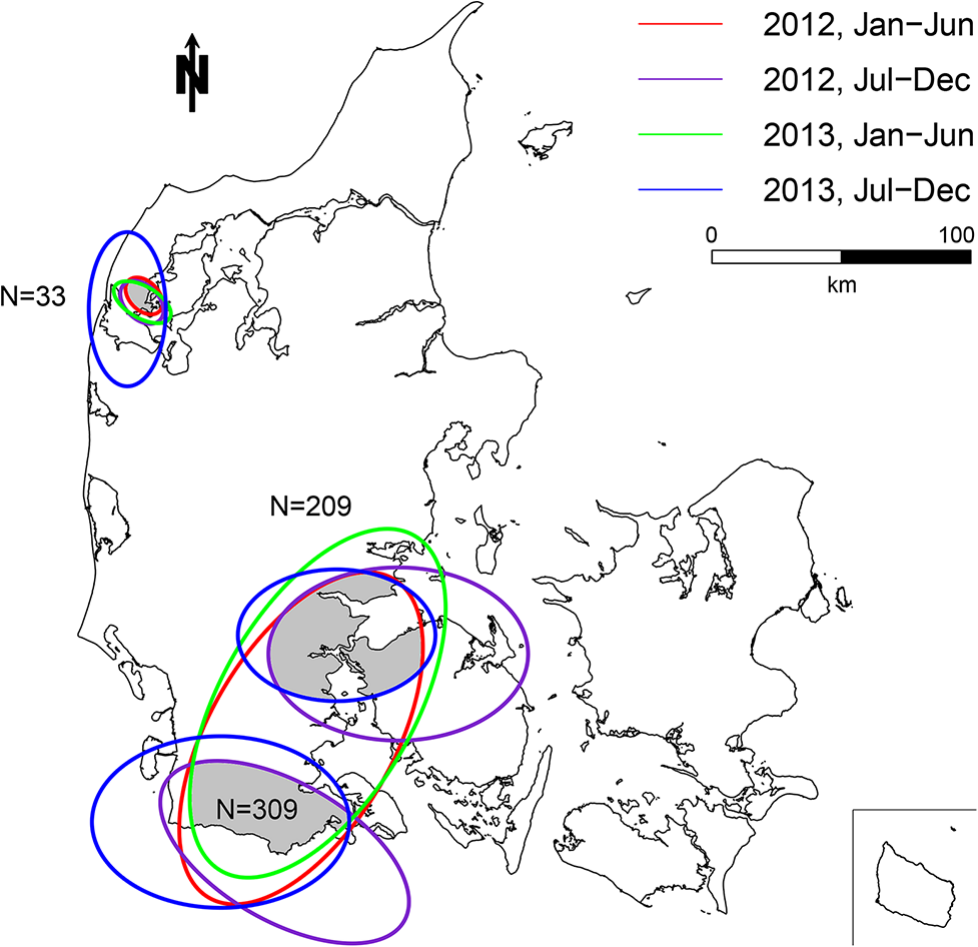
Map of the multivariate persistent clusters. Each ellipse illustrates a significant cluster (p<0.05) in one of the four time periods. The persistent clusters include a total of 551 farms situated in three distinct geographical areas. N indicates the number of farms inside each of the persistent cluster areas.

The multivariate analysis resulted in three persistent clusters, including 33, 209 and 309 farms respectively (Fig 5). In these clusters, the antimicrobial consumption was 19% higher for weaners, 104% higher for finishers, and 4% higher for sows (Table 3). Characteristics of farms inside and outside these clusters are presented in Table 4. The distribution of weaner and finisher farm sizes was significantly different inside compared to outside the clusters (Table 4).

**Table 4:** Characteristics of 6,143 farms inside versus outside the three multivariate persistent clusters.

		Farms inside clusters	Farms outside clusters	
		Actual No. (%)	Actual No (%)	p-value
Total number of farms		551 (9.0)	5,592 (91.0)	
Conventional farms		299 (54.3)	3,120 (55.8)	
Farms in the SPF register		252 (45.7)	2,472 (44.2)	0.519
**Farm type**	Nucleus farms	29 (5.3)	199 (3.6)	
	Production farms	522 (94.7)	5,393 (96.4)	0.057
**Production type**	Farrow-to-finisher	113 (20.5)	1,317 (23.6)	
	Sows and finishers	16 (2.9)	143 (2.6)	
	Sows and weaners	32 (5.8)	256 (4.6)	
	Sows	11 (2.0)	174 (3.1)	
	Weaner-finisher	72 (13.1)	843 (15.1)	
	Weaners	24 (4.4)	229 (4.1)	
	Finishers	283 (51.4)	2,630 (47.0)	0.155
**Farm size**				
Presence of sows	Small (1–254)	40 (23.3)	473 (25.0)	
	Medium (255–670)	97 (56.4)	946 (50.1)	
	Large (> 670)	35 (20.3)	471 (24.9)	0.247
Presence of weaners	Small (1–783)	48 (19.9)	674 (25.5)	
	Medium (784–2300)	143 (59.3)	1,315 (49.7)	
	Large (> 2300)	50 (20.7)	656 (24.8)	0.016
Presence of finishers	Small (1–499)	112 (23.1)	1,230 (24.9)	
	Medium (500–1600)	226 (46.7)	2,543 (51.6)	
	Large (> 1600)	146 (30.2)	1,160 (23.5)	0.005

Three covariates (farm type, production type and farm size, in bold) were included one at a time in the multivariate scanning statistics. A chi-square test was performed to test for significant differences in the prevalence of farms inside compared to outside the clusters.

The three multivariate persistent clusters were geographically close to the three persistent clusters found in the univariate analyses. One sow farm and 99 finisher farms were included in the univariate as well as the multivariate persistent clusters, which meant that 50 sow and 58 finisher farms were included in the univariate persistent clusters, but omitted from the multivariate persistent clusters.

Adding the three covariates farm type, production type and farm size one at a time to the repeated multivariate cluster analysis reduced the number of farms inside the persistent clusters to 383 (30.5%), 417 (24.3%) and 363 (34.1%), respectively.

## Discussion

This study describes how the use of antimicrobials in indoor commercial pig farms persistently cluster in certain geographical areas compared to farms in the rest of Denmark.

Clusters of antimicrobials prescribed for sows seemed to remain more constant (Fig 4) than antimicrobials prescribed for finishers (Fig 3) or for all age groups (multivariate analyses) (Fig 5). The main indications for use of antimicrobials in sows/piglets include urogenital and limbs/joints/CNS/skin disorders [10]. In practice, these conditions are typically seen as metritis-mastitis-agalactica and arthritis, respectively [29]. Feeding and stable facilities seem to play a critical role in the prevalence of both conditions [30] and represent parameters which are not expected to change markedly over time. Contrary, a large variability was found for finishers (Fig 3) and especially weaners, where no persistent clusters were detected. Gastrointestinal disorders (*Lawsonia intracellularis*, *Brachyspira* spp. and *Escherichia coli*) and respiratory disorders (*Actinobacillus pleuropneumoniae*, *Pasteurella multocida* and *Streptococcus suis*) [10] are the primary causes of treatment for weaners and finishers. Due to the infectious origin of both conditions they typically require treatment of a high proportion of pigs. Farm density is expected to have a significant effect on the transmission of airborne pathogens, and has previously been positively correlated to an increased frequency of antimicrobial treatments for sows [11], and respiratory treatments in finisher farms [31]. In general, the density of farms included in the study population was higher in the western part of Denmark than in the eastern part (Fig 2). Especially, clusters from the multivariate analysis seemed to coincide with local regions with high farm density in the western part of Denmark. However, other factors than farm density does seem to have an additional effect on the use of antimicrobials, since regional variation in farm density did not seem to coincidence with all persistent clusters (Figs 2, 3, 4 and 5). Pathogens of highest relevance within Denmark, and which may spread between pig farms in close proximity include: *Mycoplasma hyopneumoniae*, Swine Influenza Virus, Porcine Respiratory Syndrome Virus, Porcine Circo Virus type 2 and less commonly *Actinobacillus pleuropneumoniae* [32,33,34,35,36,37,38]. However, possible transmission between neighboring farms by vectors other than air [32] increase the spectrum of transmittable pathogens. Cold and humid weather conditions favor the survival of pathogens, which is why the risk of airborne disease increases during winter and may explain some of the seasonal variation in antimicrobial use (Table 2).

Our aim was to identify persisting clusters regardless of seasonal variation. The seasonal variation in antimicrobial use (Table 2) supports the choice of study design where repeated spatial analyses were performed instead of a single spatio-temporal analysis. Clusters from the univariate and multivariate analyses were located in the same geographical areas, but did not entirely overlap. Unexpectedly, only 2% (1/51) of the farms with sows, and 63% (99/157) of the finisher farms were included in both a univariate and multivariate persistent cluster. One possible reason for the rest of the farms being omitted from the multivariate persistent cluster analysis could be a lower use of antimicrobials in the two other age groups.

In the repeated multivariate analysis, finishers seem to be the age group that influences clustering the most (Table 3), due to a difference of more than 104% in antimicrobial use inside versus outside the clusters. By comparison, antimicrobial use in weaners and sows differed by 19% and 4%, respectively. Data revealed that almost half the Danish farms (45.5%) hold more than one age group of pigs at the farm. Thus, comparing the total antimicrobial consumption between farms is ideally done using a multivariate analysis to determine consistency between all sections of antimicrobials consumed. The advantage of the multivariate cluster analysis is the inclusion of all three datasets, and consequently a higher study power [23].

Three covariates addressing farm characteristics (farm type, production type and farm size) were added to the multivariate analysis separately. All of the covariates reduced the size of significant persistent clusters and therefore seem to explain some of the persistent clustering. Firstly, farm type (nucleus /production) affected the degree of persistent clustering. Table 4 indicates a borderline significant higher proportion nucleus farms inside (5.3%) compared to outside (3.6%) the persistent clusters. The limited number of nucleus farms complicates identification of significance. However, nucleus sow farms may tend to produce high quality pigs with a lower threshold for initiating treatment. In agreement with this, Nielsen et al. [31] found SPF finisher farms to have three times higher treatment frequency than non-SPF farms.

Secondly, the covariate production type includes the presence of more age groups at the farm and is expected to affect the antimicrobial use directly, because a drug prescribed for one age group may in practice be used for another, as well as indirectly, because farms with more age groups are presumed to have more restrictions on the import of pigs and therefore pathogens. Farrow-to-finisher farms have been associated with a lower use of antimicrobials in prior studies [12,11], which might be explained by the lack of movement and mixing of pigs from various farms.

Thirdly, herd size seems to influence the clustering of antimicrobials. A significant difference was observed between the distribution of weaner and finisher farm sizes inside compared to outside the persistent clusters (Table 4). Additionally, adding herd size as predictor to the scanning statistics reduced the persistent clusters (Table 3).

Inclusion of information about the prescribing veterinarian was available in the data and would be of interest to explore. Veterinarians prescribe the drugs and guide the farmer in their correct usage, and are therefore expected to affect the overall use of antimicrobials at the farm to a large extent. Furthermore, veterinarians are expected to practice in certain geographic areas and might therefore explain some of the persistent clustering. However, the veterinarians form a hierarchical structure which cannot be included in the scan statistic analysis (at the current state of the methodology). The hierarchical structure is further complicated by the fact that farms may be associated with several veterinary clinics during the study period. Therefore, analysis incorporating the hierarchical structure was considered beyond the scope of the present study.

Results from this analysis indicate how multiple factors influence the use of antimicrobials for pigs. This study indicates that farm density, farm type, production type and farm size may explain some of the clustering of antimicrobial use. However, to quantify the effect of these factors, alternative study techniques are required.

## Conclusion

This study revealed the presence of persistent clusters with higher levels of antimicrobials prescribed for finishers (157 farms), sows (51 farms) or all three age groups of pigs (551 farms). The persistent clusters were found in the same areas and overlapped to some extent. Production type, farm type and farm size all seemed to explain some of the persistent clustering in the multivariate cluster analysis, reducing the clusters by 24.3%, 30.5% and 34.1%, respectively.
